# Socioeconomic inequalities in smoking in The Netherlands before and during the Global Financial Crisis: a repeated cross-sectional study

**DOI:** 10.1186/s12889-015-1782-6

**Published:** 2015-05-06

**Authors:** Fiona E Benson, Mirte AG Kuipers, Vera Nierkens, Jan-Willem Bruggink, Karien Stronks, Anton E Kunst

**Affiliations:** Department of Public Health, Academic Medical Centre, University of Amsterdam, Meibergdreef 9, 1105 AZ Amsterdam, The Netherlands; Department of Public Health and First Line Medicine, LUMC, Albinusdreef 2, 2333 ZA Leiden, The Netherlands; Statistics Netherlands, CBS-weg 11, 6412 EX Heerlen, The Netherlands

**Keywords:** Smoking, Socioeconomic status, Inequalities, Economic recession

## Abstract

**Background:**

The Global Financial Crisis (GFC) increased levels of financial strain, especially in those of low socioeconomic status (SES). Financial strain can affect smoking behaviour.

This study examines socioeconomic inequalities in current smoking and smoking cessation in The Netherlands before and during the Global Financial Crisis (GFC).

**Methods:**

Participants were 66,960 Dutch adults (≥18 years) who took part in the annual national Health Survey (2004–2011). Period was dichotomised: ‘pre-’ and ‘during-GFC’. SES measures used were income, education and neighbourhood deprivation. Outcomes were current smoking rates (smokers/total population) and smoking cessation ratios (former smokers/ever smokers). Multilevel logistic regression models controlled for individual characteristics and tested for interaction between period and SES.

**Results:**

In both periods, high SES respondents (in all indicators) had lower current smoking levels and higher cessation ratios than those of middle or low SES. Inequalities in current smoking increased significantly in poorly educated adults of 45–64 years of age (Odds Ratio (OR) low educational level compared with high: 2.00[1.79-2.23] compared to pre-GFC 1.67[1.50-1.86], *p* for interaction = 0.02). Smoking cessation inequalities by income in 18–30 year olds increased with borderline significance during the GFC (OR low income compared to high income: 0.73[0.58-0.91]) compared to pre-GFC (OR: 0.98[0.80-1.20]), *p* for interaction = 0.051).

**Conclusions:**

Overall, socioeconomic inequalities in current smoking and smoking cessation were unchanged during the GFC. However, current smoking inequalities by education, and smoking cessation inequalities by income, increased in specific age groups. Increased financial strain caused by the crisis may disproportionately affect smoking behaviour in some disadvantaged groups.

## Background

In Dahlgren & Whitehead’s social model of health, general socioeconomic conditions are one of the population-level determinants of health [1]. General socioeconomic conditions changed in many European countries after the global economy went into recession in 2008 [2,3]. This Global Financial Crisis (GFC) led to decreased public spending in many European countries [4], and to large increases in unemployment in EU member states [5]. Economic downturns, with the resulting budgetary measures and job losses disproportionately affect those of low socioeconomic status (SES) [6], putting them under increased levels of financial strain. This financial strain may impact on other aspects of their lives, such as individual lifestyle factors, including smoking behaviour.

Financial strain can affect an individual’s smoking behaviour through a mechanism described as tension reduction [7,8], which was originally developed to explain the impact of tension on alcohol use. According to this theory individuals attempt to ameliorate the effects of feeling anxiety by more frequently enacting behaviours which give temporary relief. Support for this hypothesis with regard to smoking was given by a recent longitudinal study which found that 65–74 year old male adults and those of low educational level were more likely to smoke under increased financial strain [9]. Personal financial strain also affects cessation rates; a US study, found that in Latinos, African Americans and Caucasians of predominantly low SES, smoking cessation was less likely at 26 weeks in those with higher financial strain at baseline [10].

Financial strain on a population level may also affect smoking behaviour, however, current research provides a mixed picture. One study found that during the period 1987–2000 in the US, temporary economic downturns led to a decrease in smoking [11]. In contrast, another found that smoking prevalence in Italy had increased in 2009 compared with 2008 possibly due to the GFC, largely due to relapsing former smokers [12]. The effects of population-level financial strain on smoking can also differ amongst those of differing SES levels, sometimes increasing inequalities. However, the evidence for this is currently scant with no clear-cut agreement between studies. Firstly, Gallus et al. [13] found that in the US population there was a decrease in current smoking in the employed and an increase in the unemployed. As a result, the overall smoking prevalence changed little, but there were changes in inequalities in smoking during this period [13]. Secondly, there were overall population reductions in smoking in Iceland as a result of the GFC [14]. However, inequalities in smoking decreased as men who had experienced a reduction in income were less likely to relapse, while those whose income had increased were more likely to do so [15].

In this study, we will use data from The Netherlands from the period 2004–2011. While the effect of the GFC on the population was more dramatic in some other European countries (e.g. Ireland and Spain) during this period [16], it was felt in The Netherlands. The Netherlands entered recession [17] during the fourth quarter of 2008 [18]. The annual unemployment rate increased from 3.1 in 2008 to 4.4 in 2011, and again to 6.7 in 2013 [19]. Also, the percentage of the total population with a decrease in purchasing power increased during the GFC, from an average of 42% in the period 2004–2007 to 48% in the period 2008–2011 [20]. This worsened during the GFC from 42% in 2008 to 54% in 2011 [20].

The general aim of this paper was to examine inequalities in smoking in The Netherlands, before and during the GFC. The specific aim was to assess whether the pattern and magnitude of inequalities in current smoking and smoking cessation by education level, income level, and level of area deprivation changed after The Netherlands entered the GFC.

The immediate effects of the GFC were to increase male unemployment in Europe, due to segregation of the workforce (e.g. more men work in indicator industries such as construction) [21]. Also, there was evidence that younger, working age people were hardest hit by a downturn in employment in Europe, while their older working age colleagues suffered a less sharp rise in unemployment [22]. Such developments might increase stress particularly in these harder hit groups and could possibly lead to changes in smoking behaviour in these specific groups. Therefore, analyses were performed for subgroups of age and gender separately.

## Methods

Cross-sectional data were obtained from the annual national Health Survey (HS) for the years 2004–2011. Respondents <18 years of age were excluded (n = 22,175), as well as respondents with missing data on smoking (n = 135), which resulted in a total study population of 66,960 individuals. The HS is a continuous cross-sectional survey of residents of private homes. It aims to give an overview of the health status, healthcare usage and preventive behaviours of the Dutch population. From 2004–2009 face-to-face interviews were conducted. In 2010–2011 respondents were asked to participate by internet. In these years non-responders were approached for telephone interviews should a phone number be available, or for face-to-face interviews if not [23]. According to the Medical Ethics Review Committee of the AMC (reference number W14_143 no. 14.170180), medical ethical approval was not required for this study because it does not fall within the scope of the Dutch Medical Research (Human Subjects) Act because the participants were not subjected to any intervention or treatment. Informed consent from participants was not required for use of the existing database for this study.

Period was dichotomised as ‘pre-GFC’ (begin 2004 – end of the third quarter 2008) and ‘during-GFC’ (fourth quarter 2008–end 2011).

Two outcome variables were used: current smoking rates defined as ‘smokers/total population’ and smoking cessation ratios defined as ‘former smokers/ever smokers’. Respondents were asked two questions: 1) ‘Do you sometimes smoke?’ (yes/no) and, amongst those answering ‘no’ 2) ‘Did you smoke cigarettes in the past?’. Smokers answered ‘yes’ to question 1. Former smokers answered ‘yes’ to question 2. Ever smokers answered yes to either question.

Three measures of SES were used: standardised disposable household income, educational level and neighbourhood deprivation. Household equivalent income was measured as net household income in euros. Income information at the level of individual respondents was gained from the national tax registry. Income was split into tertiles for each of the two periods, with the ‘middle’ income level for each period respectively being: pre-GFC €17,200-€24,298 and during-GFC €19,400-€27,428. Highest completed educational level was split into three levels where ‘low’ comprised no education through to lower secondary education, ‘middle’ comprised upper secondary and middle vocational education, and ‘high’ comprised higher vocational and tertiary education. Neighbourhoods were split into three categories of deprivation (‘Most-deprived’ (83 postcode areas distributed over 40 neighbourhoods), ‘Next-most-deprived’ (252 postcode areas distributed over 100 neighbourhoods), and ‘Non-deprived’ (3276 postcode areas distributed over all remaining neighbourhoods)), based on a categorisation of the Dutch government [24]. From mid-2008, the ‘Most-deprived’ neighbourhoods were subject to a major urban renovation initiative [25]. When analysing each of the SES measures, we excluded individuals whose data on that specific measure were missing. Those excluded from analysis of each measure were: income (1144), education (2237) and neighbourhood deprivation (151). In the analysis of current smoking, 4807 individuals were missing, leaving a total of 62,153 individuals, and in the analysis of smoking cessation a total of 39,804 individuals (‘ever smokers’) were included in the analysis.

Some other individual characteristics were also used and were defined as follows. Age was separated into four groups: young adults (‘18–30 years’), working age (‘31-44 years’ and ‘45–64 years’), and pensioners (‘>64 years’). Ethnicity was taken from the national continuous population registry. This was separated into three groups based on the definition of Statistics Netherlands: ‘Western minorities’ (individuals without two Dutch parents, who were born in Europe (excluding Turkey), North America, Oceania, Indonesia or Japan)) [26], ‘non-Western minorities’ (individuals without two Dutch parents, who were born in Africa, Latin America, Asia (excluding Indonesia or Japan) or Turkey) [27], and ‘ethnic Dutch’ (individuals with both parents born in The Netherlands, irrespective of the participant’s own country of birth) [28]. Household composition was separated into the five categories: ‘Couple, with children’; ‘Couple, without children’; ‘Single, with children’; ‘Single without children’; and, ‘Other’, for all other groups.

In the statistical analyses we applied multilevel logistic regression models containing a random intercept at the neighbourhood level. Current smoking and smoking cessation were the dependent variables. Model 1 included the following predictors: time (in years), period (pre- and during-GFC), age (in the three distinguished age groups), gender, ethnicity, and household composition. Time was included to control for secular trends in smoking. In a sensitivity analysis, in which we did not control for time, we found essentially the same results as those reported below. Model 2 also included educational level, income, and area deprivation. Associations are presented for the periods before and during the GFC. Interaction between period and all covariates was tested by means of interaction terms in a model that includes all covariates. Analyses were performed for subgroups of age and gender separately and three-way interactions were tested (e.g. education level *period *gender).

## Results

**Figure 1:**
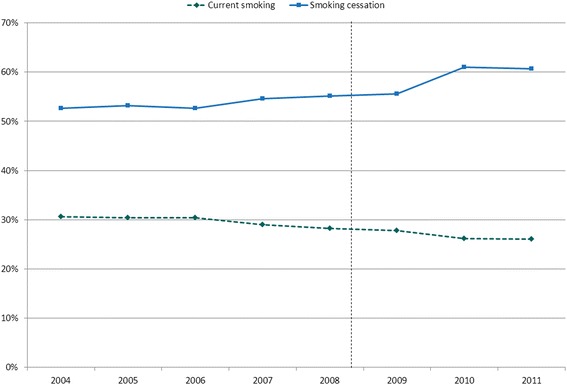
Trends in current smoking and smoking cessation.

Respondents pre-GFC and during the GFC had similar characteristics (Table 1). The trend in the total population is towards decreasing current smoking and increasing smoking cessation (Figure 1).

**Table 1:** **Descriptive information, smoking prevalence and quit ratio for the study population pre- and during-GFC**

	**Descriptive information**		**Smoking prevalence**		**Smoking cessation**	
	**Pre-GFC**	**During-GFC**	**Pre-GFC**	**During-GFC**	**Pre-GFC**	**During-GFC**
N	34,981	31,979				
Age (mean, SD)	48.0 ± 17.4	48.9 ± 17.5				
	% of respondents					
**Total population**	-	-	29.9	26.5	53.5	59.5
**Gender**						
Male	48.3	48.5	33.8	29.8	52.1	58.8
Female	51.7	51.5	26.3	23.5	55.0	60.4
**Age**						
18 – 30 years of age	18.1	18.0	36.1	33.6	32.4	38.6
31 – 44 years of age	26.7	24.0	32.9	29.3	43.7	49.6
45 – 64 years of age	35.9	37.6	30.9	27.8	57.9	62.2
>64 years of age	19.2	20.4	18.0	14.8	72.5	79.4
**Ethnicity**						
Ethnic Dutch	85.7	89.5	29.4	26.4	54.8	60.4
Western Minorities	8.2	7.3	33.8	26.8	49.9	60.9
Non-Western Minorities	6.1	3.2	32.1	27.9	34.8	43.1
**Household composition**						
Couple, with children	41.6	38.8	29.7	26.3	50.7	55.9
Couple, no children	35.1	36.3	26.3	22.5	62.3	69.0
Single, with children	17.2	18.3	33.7	30.6	45.9	53.0
Single, no children	4.9	4.7	41.8	38.9	35.5	41.0
Other	1.3	1.9	37.9	37.7	38.5	40.6
**Education level**						
High	23.0	27.5	22.0	19.1	62.4	68.3
Middle	37.2	32.6	31.0	28.3	51.8	57.0
Low	39.8	39.9	33.4	30.2	50.5	57.2
**Household income**						
High	33.3	33.3	25.2	21.7	60.4	66.0
Middle	33.4	33.4	30.4	27.5	53.5	57.8
Low	33.3	33.3	33.4	30.4	49.7	52.8
**Neighbourhood deprivation**						
Non-deprived	86.8	87.5	29.4	25.8	54.3	60.6
Next-most-deprived (100 areas)	9.4	8.9	33.0	30.7	49.2	54.9
Most-deprived (40 areas)	3.8	3.5	34.5	34.1	45.9	45.9

Smoking prevalence decreased and smoking cessation increased during the GFC (Table 1). This was observed for all subgroups of gender, age, ethnicity, household composition, educational level and income. The single exception was that the quit ratio was unchanged in the Most-deprived areas.

In both periods, there are substantial inequalities in smoking according to each SES indicator. Those of high SES smoke less than those of middle SES, who in turn smoke less than those of low SES (Table 1). The pattern seen for smoking cessation is similar but in the opposite direction, with an increase in quit ratios with increasing SES (Table 1). Smoking prevalence is also seen to decrease and smoking cessation to increase with increasing age (Table 1).

Regression based estimates of associations of current smoking and smoking cessation with covariates are shown in Table 2. In model 2, strong independent associations are observed for all socio-demographic variables, including the three socioeconomic variables. Differences according to educational level are larger than those according to income and area deprivation. There is an association with the GFC (current smoking (Odds Ratio (OR): 0.96 (0.89-1.03)) and smoking cessation (OR: 1.08 (1.0-1.18))) which persists after controlling for all covariates (OR(95%CI): current smoking (0.97 (0.90-1.04)) and smoking cessation (OR: 1.08 (0.99-1.18))), though without statistical significance. Current smoking decreases in a graduated manner as age group increases. Smoking cessation increases in a graduated manner as age group increases, with those of >64 years of age having quit ratios significantly larger than those of 31–33 or 45–64 years of age (OR(95%CI): >64 (7.17(6.60-7.80)) compared with 31–44 (1.53(1.43-1.65)) and 45–64 (2.66(2.49-2.85))).

**Table 2:** **Association between covariates and current smoking and smoking cessation in a multilevel logistic regression model**

	**Current smoking**		**Smoking cessation**	
	**Model 1**	**Model 2**	**Model 1**	**Model 2**
**Time (in years)**	0.97 [0.96-0.98]	0.97 [0.96-0.99]	1.04 [1.02-1.06]	1.04 [1.02-1.06]
**Time period**				
Pre-GFC	1.00	1.00	1.00	1.00
During-GFC	0.96 [0.89-1.03]	0.97 [0.90-1.04]	1.08 [1.00-1.18]	1.08 [0.99-1.18]
**Age**				
18 – 30 years of age	1.00	1.00	1.00	1.00
31 – 44 years of age	0.90 [0.86-0.95]	0.89 [0.85-0.94]	1.50 [1.40-1.60]	1.53 [1.43-1.65]
45 – 64 years of age	0.80 [0.76-0.84]	0.75 [0.71-0.79]	2.54 [2.39-2.70]	2.66 [2.49-2.85]
>64 years of age	0.34 [0.32-0.37]	0.26 [0.24-0.28]	5.89 [5.45-6.37]	7.17 [6.60-7.80]
**Gender**				
Male	1.00	1.00	1.00	1.00
Female	0.70 [0.68-0.73]	0.66 [0.64-0.69]	1.21 [1.16-1.26]	1.26 [1.20-1.31]
**Ethnicity**				
Ethnic Dutch	1.00	1.00	1.00	1.00
Western minorities	1.12 [1.05-1.20]	1.10 [1.02-1.17]	0.92 [0.86-1.00]	0.94 [0.87-1.02]
Non-western minorities	0.88 [0.81-0.95]	0.70 [0.64-0.77]	0.69 [0.62-0.77]	0.82 [0.72-0.92]
**Household composition**				
Couple, with children	1.00	1.00	1.00	1.00
Couple, no children	1.11 [1.06-1.16]	1.15 [1.10-1.21]	1.03 [0.98-1.09]	1.01 [0.96-1.06]
Single, with children	1.68 [1.60-1.77]	1.66 [1.57-1.75]	0.55 [0.51-0.58]	0.56 [0.53-0.60]
Single, no children	1.93 [1.79-2.10]	1.80 [1.65-1.95]	0.50 [0.46-0.56]	0.54 [0.49-0.60]
Other	1.63 [1.42-1.86]	1.61 [1.38-1.87]	0.61 [0.51-0.73]	0.59 [0.49-0.72]
**Education level**				
High		1.00		1.00
Middle		1.57 [1.49-1.65]		0.70 [0.66-0.74]
Low		2.14 [2.04-2.26]		0.52 [0.49-0.55]
**Household income**				
High		1.00		1.00
Middle		1.19 [1.14-1.25]		0.86 [0.82-0.91]
Low		1.34 [1.27-1.40]		0.76 [0.72-0.81]
**Neighbourhood deprivation**				
Non-deprived		1.00		1.00
Next-most-deprived		1.22 [1.15-1.31]		0.86 [0.80-0.93]
Most-deprived		1.26 [1.13-1.40]		0.84 [0.74-0.95]

In Table 3 we examined the association between covariates and smoking outcomes before and during the GFC. In most cases, associations of socio-demographic variables with smoking outcomes remained similar. One exception is that male–female differences in smoking cessation significantly declined during the GFC (ORs decreased from 1.32 to 1.18, *p-*value for the interaction = 0.009). Smoking prevalence in western minorities compared with the ethnic Dutch significantly decreased and smoking cessation significantly increased during the GFC compared with pre-GFC (OR(95%CI): smoking prevalence: 1.17(1.08-1.28) to 0.99(0.89-1.10) and smoking cessation: 0.85(0.76-0.94) to 1.10(0.98-1.24)). Patterns of inequalities tended to increase between the two time periods, especially between Non-deprived and the Most-deprived neighbourhoods, though without statistical significance.

**Table 3:** **Association between covariates and current smoking and smoking cessation pre- and during-GFC**

	**Current smoking**			**Smoking cessation**		
	**Pre-GFC**	**During-GFC**	***p*** **-value for interaction [a]**	**Pre-GFC**	**During-GFC**	***p*** **-value for interaction [a]**
**Time (in years)**	0.97 [0.96-0.99]	0.97 [0.89-1.05]	0.934	1.02 [1.00-1.04]	1.09 [1.05-1.13]	0.001
**Age**						
18 – 30 years of age	1.00	1.00		1.00	1.00	
31 – 44 years of age	0.90 [0.84-0.97]	0.89 [0.81-0.97]	0.817	1.60 [1.45-1.76]	1.46 [1.31-1.62]	0.209
45 – 64 years of age	0.76 [0.71-0.81]	0.74 [0.69-0.80]	0.688	2.81 [2.57-3.08]	2.49 [2.25-2.75]	0.068
>64 years of age	0.27 [0.25-0.30]	0.25 [0.22-0.27]	0.085	6.94 [6.23-7.73]	7.49 [6.64-8.44]	0.336
**Gender**						
Male	1.00	1.00		1.00	1.00	
Female	0.65 [0.62-0.68]	0.69 [0.65-0.73]	0.090	1.32 [1.25-1.40]	1.18 [1.11-1.26]	0.009
**Ethnicity**						
Ethnic Dutch	1.00	1.00		1.00	1.00	
Western minorities	1.17 [1.08-1.28]	0.99 [0.89-1.10]	0.014	0.85 [0.76-0.94]	1.10 [0.98-1.24]	0.001
Non-western minorities	0.70 [0.63-0.78]	0.70 [0.60-0.82]	0.957	0.79 [0.68-0.92]	0.86 [0.70-1.06]	0.484
**Household composition**						
Couple, with children	1.00	1.00		1.00	1.00	
Couple, no children	1.18 [1.11-1.25]	1.12 [1.05-1.20]	0.479	0.97 [0.90-1.03]	1.07 [0.99-1.15]	0.937
Single, with children	1.71 [1.59-1.84]	1.60 [1.48-1.73]	0.290	0.52 [0.48-0.57]	0.61 [0.55-0.67]	0.562
Single, no children	1.76 [1.57-1.96]	1.85 [1.63-2.10]	0.255	0.55 [0.47-0.63]	0.53 [0.46-0.62]	0.417
Other	1.51 [1.21-1.89]	1.68 [1.37-2.06]	0.741	0.60 [0.45-0.80]	0.59 [0.46-0.77]	0.978
**Education level**			0.351			0.535
High	1.00	1.00		1.00	1.00	
Middle	1.54 [1.44-1.64]	1.61 [1.49-1.74]	0.354	0.72 [0.66-0.78]	0.68 [0.62-0.74]	0.347
Low	2.09 [1.95-2.24]	v2.20 [2.05-2.37]	0.305	0.53 [0.49-0.58]	0.51 [0.47-0.55]	0.449
**Household income**			0.939			0.252
High	1.00	1.00		1.00	1.00	
Middle	1.17 [1.10-1.24]	1.23 [1.15-1.32]	0.254	0.89 [0.83-0.96]	0.83 [0.77-0.90]	0.174
Low	1.34 [1.25-1.42]	1.34 [1.25-1.44]	0.897	0.78 [0.73-0.84]	0.73 [0.68-0.80]	0.246
**Neighbourhood deprivation**			0.210			0.383
Non-deprived	1.00	1.00		1.00	1.00	
Next-most-deprived	1.20 [1.10-1.30]	1.26 [1.14-1.39]	0.414	0.86 [0.78-0.94]	0.86 [0.77-0.97]	0.935
Most-deprived	1.21 [1.06-1.38]	1.33 [1.14-1.55]	0.316	0.89 [0.76-1.04]	0.77 [0.64-0.93]	0.230

[a] The p-value at the same row as the variable name refers to the test on the overall interaction between period and this variable (measured on a continuous scale).

In Table 4 we examine changes in the association between SES variables and smoking outcomes respectively for adults in four age groups. Overall, in income and education, inequalities in smoking were stable or tended to increase between periods. Changes in smoking inequalities as a result of the GFC were very similar in all age groups. This was confirmed by three-way interaction tests (e.g. education level*period*age), in which we found that none of the interactions were statistically significant. Among respondents aged 18–30 years, income-related inequalities in smoking cessation were borderline significantly larger during the GFC than pre-GFC (OR(95%CI): 18–30 years pre-GFC: 0.98(0.80-1.20) and during-GFC: 0.73 (0.58-0.91), p = 0.051). In those of 45–64 years of age, those of low income had significantly less decrease in current smoking during the GFC compared with pre-GFC (OR(95%CI): pre-GFC 1.67 (1.50-1.86) and during GFC: 2.00 (1.79-2.23) p = 0.02).

**Table 4:** Pre- and during-GFC associations between SES variables and current smoking and smoking cessation by age-group*

	**Current smoking**			**Smoking cessation**		
	**Pre-GFC**	**During-GFC**	***p*** **interaction** ^**a**^	**Pre-GFC**	**During-GFC**	***p*** **interaction** ^**a**^
**18 – 30 years of age**						
**Education level**			0.341			0.939
High	1.00	1.00	Ref	1.00	1.00	Ref
Middle	1.88 [1.61-2.21]	1.93 [1.63-2.28]	0.843	0.61 [0.49-0.75]	0.63 [0.51-0.78]	0.823
Low	3.18 [2.67-3.79]	3.57 [2.94-4.32]	0.371	0.33 [0.26-0.43]	0.33 [0.25-0.43]	0.945
**Household income**			0.558			0.047
High	1.00	1.00	Ref	1.00	1.00	Ref
Middle	1.09 [0.95-1.25]	1.18 [1.00-1.39]	0.474	0.92 [0.76-1.12]	0.99 [0.80-1.24]	0.611
Low	1.24 [1.08-1.44]	1.33 [0.96-1.30]	0.542	0.98 [0.80-1.20]	0.73 [0.58-0.91]	0.051
**Neighbourhood deprivation**			0.428			0.289
Non-deprived	1.00	1.00	Ref	1.00	1.00	Ref
Next-most-deprived	1.24 [1.00-1.53]	1.02 [0.82-1.26]	0.196	0.92 [0.68-1.25]	1.08 [0.81-1.44]	0.457
Most-deprived	1.10 [0.82-1.49]	1.08 [0.78-1.49]	0.917	0.72 [0.44-1.18]	0.95 [0.59-1.51]	0.418
**31 – 44 years of age**						
**Education level**			0.931			0.890
High	1.00	1.00	Ref	1.00	1.00	Ref
Middle	1.73 [1.54-1.95]	1.80 [0.58-2.06]	0.654	0.62 [0.54-0.72]	0.60 [0.52-0.71]	0.789
Low	2.84 [2.49-3.24]	2.82 [2.44-3.27]	0.950	0.43 [0.36-0.50]	0.43 [0.36-0.52]	0.892
**Household income**			0.156			0.481
High	1.00	1.00	Ref	1.00	1.00	Ref
Middle	1.33 [1.18-1.50]	1.24 [1.08-1.43]	0.468	0.86 [0.74-1.00]	0.85 [0.72-1.00]	0.867
Low	1.59 [1.40-1.80]	1.39 [1.21-1.53]	0.157	0.72 [0.62-0.84]	0.77 [0.65-0.85]	0.513
**Neighbourhood deprivation**			0.246			0.400
Non-deprived	1.00	1.00	Ref	1.00	1.00	Ref
Next-most-deprived	1.30 [1.12-1.49]	1.48 [1.23-1.78]	0.267	0.76 [0.64-0.91]	0.71 [0.57-0.89]	0.661
Most-deprived	1.25 [1.02-1.53]	1.41 [1.06-1.87]	0.491	0.97 [0.75-1.24]	0.82 [0.58-1.16]	0.450
**45 – 64 years of age**						
**Education level**			0.017			0.099
High	1.00	1.00	Ref	1.00	1.00	Ref
Middle	1.34 [1.20-1.50]	1.46 [1.30-1.65]	0.310	0.80 [0.71-0.90]	0.73 [0.64-0.83]	0.324
Low	1.67 [1.50-1.86]	2.00 [1.79-2.23]	0.020	0.65 [0.58-0.73]	0.56 [0.50-0.64]	0.095
**Household income**			0.487			0.466
High	1.00	1.00	Ref	1.00	1.00	Ref
Middle	1.20 [1.10-1.32]	1.32 [1.19-1.46]	0.184	0.84 [0.76-0.93]	0.75 [0.67-0.84]	0.145
Low	1.43 [1.29-1.59]	1.50 [1.34-1.67]	0.557	0.67 [0.60-0.75]	0.64 [0.57-0.72]	0.562
**Neighbourhood deprivation**			0.322			0.474
Non-deprived	1.00	1.00	Ref	1.00	1.00	Ref
Next-most-deprived	1.20 [1.04-1.38]	1.34 [1.15-1.57]	0.291	0.83 [0.71-0.97]	0.79 [0.66-0.94]	0.638
Most-deprived	1.32 [1.04-1.67]	1.43 [1.10-1.84]	0.650	0.80 [0.61-1.04]	0.71 [0.53-0.95]	0.573
**>64 years of age**						
**Education level**			0.771			0.829
High	1.00	1.00	Ref	1.00	1.00	Ref
Middle	1.24 [0.99-1.56]	1.14 [0.89-1.46]	0.622	0.89 [0.70-1.12]	0.85 [0.65-1.13]	0.838
Low	1.26 [1.02-1.56]	1.19 [0.95-1.48]	0.673	0.76 [0.61-0.95]	0.77 [0.60-0.98]	0.933
**Household income**			0.445			0.086
High	1.00	1.00	Ref	1.00	1.00	Ref
Middle	1.12 [0.92-1.35]	1.20 [0.97-1.47]	0.626	0.92 [0.75-1.12]	0.74 [0.59-0.79]	0.160
Low	1.20 [1.00-1.44]	1.33 [1.09-1.62]	0.434	0.81 [0.67-0.99]	0.63 [0.50-0.79]	0.066
**Neighbourhood deprivation**			0.120			0.168
Non-deprived	1.00	1.00	Ref	1.00	1.00	Ref
Next most deprived	0.99 [0.80-1.24]	1.13 [0.88-1.45]	0.447	1.07 [0.85-1.36]	1.08 [0.82-1.43]	0.949
Most-deprived	0.99 [0.70-1.40]	1.44 [0.97-2.12]	0.151	1.10 [0.75-1.59]	0.66 [0.43-1.00]	0.068

*Controlled for time (in years), gender, ethnicity and household composition.

^a^ *p*-values in the same row as the variable name refer to the test on the overall interaction between period and this variable (measured on a continuous scale).

In Table 5 we examined changes in the association between SES variables and smoking outcomes for males and females. Changes in smoking inequalities as a result of the GFC were very similar for males and females. This was confirmed by three-way interaction tests (e.g. education level*period*age), in which we found that none of the interactions were statistically significant. However, we observed that in females there was a tendency, although not statistically significant, towards widening inequalities in current smoking and smoking cessation in Most-deprived compared with Non-deprived areas. This non-significant trend was also observed in smoking cessation in males during the GFC.

**Table 5:** Pre- and during-GFC associations between SES variables and current smoking and smoking cessation by gender*

	**Current smoking**			**Smoking cessation**		
	**Pre-GFC**	**During-GFC**	**P-value for interaction** ^**a**^	**Pre-GFC**	**During-GFC**	**P-value for interaction** ^**a**^
**Males**						
**Education level**			0.914			0.601
High	1.00	1.00	Ref	1.00	1.00	Ref
Middle	1.46 [1.33-1.59]	1.59 [1.44-1.75]	0.177	0.75 [0.68-0.84]	0.70 [0.63-0.79]	0.392
Low	2.00 [1.83-2.20]	2.05 [1.85-2.26]	0.727	0.60 [0.54-0.67]	0.78 [0.72-0.85]	0.534
**Household income**			0.719			0.445
High	1.00	1.00	Ref	1.00	1.00	Ref
Middle	1.16 [1.06-1.26]	1.16 [1.05-1.27]	0.993	0.90 [0.82-0.99]	0.91 [0.81-1.01]	0.930
Low	1.32 [1.21-1.44]	1.29 [1.17-1.43]	0.718	0.80 [0.72-0.89]	0.76 [0.68-0.85]	0.440
**Neighbourhood deprivation**			0.258			0.344
Non-deprived	1.00	1.00	Ref	1.00	1.00	Ref
Next-most-deprived	1.11 [0.99-1.25]	1.29 [1.13-1.47]	0.097	0.86 [0.75-0.99]	0.78 [0.67-0.91]	0.351
Most-deprived	1.16 [0.97-1.40]	1.20 [0.97-1.47]	0.824	0.88 [0.70-1.10]	0.80 [0.63-1.02]	0.606
**Females**						
**Education level**			0.277			0.955
High	1.00	1.00	Ref	1.00	1.00	Ref
Middle	1.67 [1.50-1.85]	1.66 [1.48-1.86]	0.941	0.67 [0.59-0.75]	0.66 [0.58-0.75]	0.865
Low	2.30 [2.07-2.55]	2.45 [2.19-2.74]	0.386	0.47 [0.42-0.53]	0.47 [0.41-0.53]	0.923
**Household income**			0.694			0.526
High	1.00	1.00	Ref	1.00	1.00	Ref
Middle	1.17 [1.07-1.28]	1.31 [1.18-1.45]	0.092	0.86 [0.77-0.95]	0.74 [0.66-0.83]	0.058
Low	1.32 [1.21-1.45]	1.37 [1.24-1.52]	0.583	0.73 [0.66-0.81]	0.69 [0.61-0.78]	0.465
**Neighbourhood deprivation**			0.450			0.860
Non-deprived	1.00	1.00	Ref	1.00	1.00	Ref
Next-most-deprived	1.30 [1.15-1.46]	1.23 [1.07-1.42]	0.565	1.14 [0.93-1.40]	0.95 [0.81-1.12]	0.221
Most-deprived	1.25 [1.04-1.50]	1.53 [1.22-1.91]	0.159	0.81 [0.58-1.14]	0.72 [0.55-0.94]	0.227

*Controlled for time (in years), age, ethnicity and household composition.

^a^ *p*-values in the same row as the variable name refer to the test on the overall interaction between period and this variable (measured on a continuous scale).

## Discussion

Inequalities in current smoking and smoking cessation were found both before and during the GFC. We cannot demonstrate a significant increase in inequalities overall, however, there was a general tendency to increasing inequalities during the GFC compared with pre-GFC. Especially in the 18–30 age group, we found evidence of almost significant widening poor-rich inequalities in smoking cessation, and in the 45–64 age group we found a significant widening in educational inequalities in current smoking.

The observed trends may not only be influenced by the GFC, but also by other factors such as tobacco control policies. Two new national smoking policies were introduced in The Netherlands during our study period in the ‘during-GFC’ period. Hospitality industry smoke-free workplace legislation, introduced in July 2008 [29], increased the number of successful quit attempts [30]. Free pharmacotherapy for individuals undertaking smoking cessation behavioural support, introduced in 2011, was followed by a minor (1%) decrease in smoking prevalence and a modest increase in the number of successful quit attempts [31,32]. In both cases socioeconomic inequalities remained unchanged [30,33], so these policies are unlikely to have been responsible for effects found in this study.

The HS dataset is based on a continuous cross-sectional survey. Survey methods were changed to ‘mixed-mode’ in 2010, at the time of our ‘during-GFC’ period. However, a Statistics Netherlands report indicated that for smoking amongst other variables, the mode effects were generally not significant [23].

The quit ratio, as used in this study, did not measure when former smokers quit, since this was not asked in the survey. The quit ratio therefore captures all former smokers, including those who have quit before the studied period and remained non-smokers since. We studied changes in the quit ratio between the two periods, within the same source population. The increase found in cessation between the two periods indicates that an additional number of people have quit smoking during the crisis. Under the assumption that the study population in both periods reflect the source population, the quit ratio was able to detect changes in smoking cessation over the two periods, but was not sensitive to the exact timing of the occurrence of change.

For many years, smoking prevalence in The Netherlands has been decreasing [34,35]. This seems to have continued during the GFC [34,35]. Such trends may have concealed an upward effect of the GFC on smoking. Furthermore, it is possible that changes in trends in the two periods might be missed by looking only at the overall prevalence in each period. However, our explorative analysis of trends within each period (Figure 1) found no evidence for an upward trend in smoking during the GFC.

For current smoking, our findings are similar to those found in the US [13], where the population prevalence also changed little after the onset of the GFC. In that study no change in inequalities by education were seen [13]. We, also, didn’t observe such an increase in the overall population, however, we observed an increase in inequalities for the age group 45–64 years.

McClure et al. [15] found an effect of the GFC on smoking behaviour in Iceland, where men whose income was reduced over the period 2007–2009 were significantly less likely to relapse than men whose income increased during the same period. This contrasts with our results, where inequalities according to income seemed to have increased for younger adults.

There were no significant changes in overall inequalities according to area deprivation, income or education in current smoking or in smoking cessation. Good social protection has been shown to mitigate some effects of economic crises on health [4,36,37]. As The Netherlands is one of the European countries spending most on social protection per inhabitant [38], it is possible that this may have buffered any effects of increased financial strain on smoking behaviour in the Dutch population.

We observed that current smoking amongst poorly educated 45–64 year olds decreased to a lesser extent during the GFC than amongst highly educated 45–64 year olds. Older workers and those of low educational level feel more job insecurity than younger and better educated workers respectively, regardless of levels of social protection available [39]. Self-perceived job insecurity increases psychological morbidity [40] and ill health [39]. Also, workers of this age group in The Netherlands may be considered by many Dutch employers to be too expensive due to higher pension contributions, absenteeism insurance and health care costs [41]. It is possible that this self-perceived vulnerability, with its effects on general stress among poorly educated adults, increased during the GFC. In line with the tension reduction mechanism [7,8], this might lead to increased current smoking amongst this group.

Among young adults, we found evidence to suggest that smoking cessation rates of those with low income increased to a lesser extent during the GFC than among their high income peers. While the percentage of young people (15 – 25 years of age) searching for a job in The Netherlands did not change greatly during the period studied [42], this may have occurred because young people’s response to the GFC may have been to remain in education or give up the search altogether [43]. The prospect of poor employment opportunities, particularly in individuals of low SES where job prospects were already more limited, might increase feelings of anxiety and lack of control. Even if employed, those in poorly-paid jobs feel more self-perceived job insecurity than those in better-paid jobs [39]. Increased anxiety during the GFC might have decreased the success of quit attempts [10].

Differences in smoking prevalence and cessation rates between Most-deprived and Non-deprived areas tended to widen during the GFC compared with pre-GFC. This tendency was particularly found in those of >30 years and in women, although no statistical differences by age were found. This widening happened despite the 40 Most-deprived areas undergoing a major urban regeneration project from 2008 onwards [25]. One possible explanation for this widening is that residents of the Most-deprived areas experienced a greater increase in anxiety and financial strain, and this influenced their smoking behaviour. A qualitative study on a deprived area in Ireland found that the GFC had a substantial effect on family and community life, which were affected by anxiety due to fear of poverty [44]. It is possible that a similar but less pronounced effects occurred in the Most-deprived areas of The Netherlands.

The effects of the GFC on inequalities in current smoking and smoking cessation are generally small. One possible explanation is that part of these effects will need more time to develop. For example, though the Netherlands went into recession during the fourth quarter of 2008, government budget cuts only started to be felt by Dutch society in 2012 [45] and only since then has there been a decrease in the purchasing power of most Dutch households, an increase in the number of unemployed and people receiving social benefit, and an increase in the number of those living in poverty [46]. This delay is reflected in our data, where income did not decrease between the periods at large. Future studies should assess the long term effects of population-level financial strain on smoking trends and inequalities in The Netherlands.

## Conclusion

While inequalities in current smoking and smoking cessation were generally stable, education-related inequalities in current smoking in 45–64 year old adults and income-related inequalities in smoking cessation in younger adults increased during the GFC compared with pre-GFC. This suggests that increased financial strain caused by the crisis may disproportionately affect smoking behaviour in some specific disadvantaged groups.
